# Attributes, Fabrication, and Applications of Gallium‐Based Liquid Metal Particles

**DOI:** 10.1002/advs.202000192

**Published:** 2020-04-22

**Authors:** Yiliang Lin, Jan Genzer, Michael D. Dickey

**Affiliations:** ^1^ Department of Chemical and Biomolecular Engineering North Carolina State University Raleigh NC 27695‐7905 USA

**Keywords:** colloids, EGaIn, gallium, liquid metals, particles

## Abstract

This work discusses the attributes, fabrication methods, and applications of gallium‐based liquid metal particles. Gallium‐based liquid metals combine metallic and fluidic properties at room temperature. Unlike mercury, which is toxic and has a finite vapor pressure, gallium possesses low toxicity and effectively zero vapor pressure at room temperature, which makes it amenable to many applications. A variety of fabrication methods produce liquid metal particles with variable sizes, ranging from nm to mm (which is the upper limit set by the capillary length). The liquid nature of gallium enables fabrication methods—such as microfluidics and sonication—that are not possible with solid materials. Gallium‐based liquid metal particles possess several notable attributes, including a metal–metal oxide (liquid–solid) core–shell structure as well as the ability to self‐heal, merge, and change shape. They also have unusual phase behavior that depends on the size of the particles. The particles have no known commercial applications, but they show promise for drug delivery, soft electronics, microfluidics, catalysis, batteries, energy harvesting, and composites. Existing challenges and future opportunities are discussed herein.

## Graphical abstract

This review discusses gallium‐based liquid metal particles. Gallium‐based liquid metals possess several unique properties including the combination of metallic and fluidic properties at room temperature. Liquid particles can be produced through a wide range of techniques that are not possible with solid metals. Such particles show promise for drug delivery, soft electronics, microfluidics, catalysis, composites, and energy harvesting.

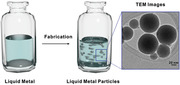

## 1 Introduction

Metals that exist as liquids at room temperature are attractive due to their unique combination of fluidic and metallic properties. As a fluid, liquid metal is the softest among all conductive materials.^[^ ^1^ ^]^ It can be deformed while maintaining metallic conductivity. The ability to remain highly conductive, yet soft and deformable, endows liquid metals with unique attributes that can be employed in numerous applications, especially in soft and stretchable electronics.^[^ ^2^ ^]^

Mercury is a commonly known liquid metal, yet its toxicity limits its application.^[^ ^3^, ^4^ ^]^ In contrast, gallium and its alloys serve as promising liquid metals due to their low toxicity and near‐zero vapor pressure.^[^ ^5^ ^]^ The native gallium oxide that forms on these metals lowers the interfacial tension of liquid metal while adding a thin, mechanical “skin” to the surface, which allows the liquid metal to sustain non‐spherical structures. The presence of oxide skin helps liquid metals to adhere to various substrates, which facilitates patterning of these liquids. Recent reviews have introduced methods to pattern liquid metals.^[^ ^6^, ^7^ ^]^ The presence of the native oxide skin enables the liquid metal to be employed in a variety of applications,^[^ ^8^ ^]^ including, stretchable wires,^[^ ^9^, ^10^ ^]^ soft conductors,^[^ ^11^, ^12^, ^13^, ^14^ ^]^ sensors,^[^ ^15^, ^16^, ^17^, ^18^, ^19^ ^]^ antennas,^[^ ^20^, ^21^, ^22^, ^23^, ^24^, ^25^, ^26^, ^27^, ^28^ ^]^ electrodes,^[^ ^29^, ^30^, ^31^, ^32^ ^]^ and circuit components.^[^ ^33^, ^34^ ^]^

Liquid metals can also serve as “reactors” to produce thin oxide species or to promote reactions with surrounding molecules.^[^ ^35^ ^]^ While the native oxide on the surface can be used for catalysis,^[^ ^36^ ^]^ the surface oxide can also be physically separated from the metal as a means of producing thin oxides at room temperature. The composition of the oxide can be modified by dissolving metals into liquid metal. Metal additives dissolved within liquid metal will form oxides at the interface under the right conditions if they have a more favorable Gibbs free energy of formation than gallium oxide. The presence of these metal “dopants” at the interface of the metal can also lower the energy necessary for reactions, such as electrochemical reduction of carbon dioxide.^[^ ^37^, ^38^ ^]^ Liquid metals can also promote free radical polymerization without the need for conventional molecular initiators.^[^ ^39^ ^]^

Liquid metal particles are easy to fabricate due to their fluidic properties, while the metallic nature endows them with useful applications, including (but not limited to) catalysts, composites, energy harvesting materials, therapeutics, solders, sensors, and electronic inks.^[^ ^40^ ^]^ This review discusses the attributes, fabrication, and recent applications of liquid metal particles.

## 2 What Is a Particle?

Typically, a volume of liquid placed on a substrate loses its spherical structure under the influence of gravity when gravitational forces exceed interfacial forces. There exists a particular length, referred to as capillary length ($\lambda_{\text{c}}=\sqrt{\frac{\gamma }{\rho g}}$, where *g* is the gravitational acceleration and ρ is the density of the fluid, and γ is the surface tension), beyond which the gravitational forces start to exceed interfacial forces. For gallium, the capillary length is ≈3 mm, assuming the oxide‐coated metal is surrounded by air and that the surface tension of the interface is equal to the value of the yield stress of the oxide; this value does not change much if the surface energy of bare metal is utilized instead.^[^ ^41^ ^]^ The capillary length becomes slightly larger if the surrounding fluid is denser than air due to buoyancy effects. In this case, the term for density, ρ, gets replaced by the difference in density between the metal and the surrounding fluid. If the surrounding fluid has a ρ ≈ 1 g mL^−1^ (typical of many aqueous and organic fluids), the capillary length increases by ≈10%. Thus, the liquid metal particles discussed within this review are smaller than a few mm in all dimensions, which effectively excludes larger volumes of metal as well as shapes—such as wires—that are long in one dimension. It does not, however, limit the review to spherical shapes.

## 3 Liquid Metals

This review limits itself to metals that are stable as liquids at or very near room temperature while excluding mercury due to its toxicity. This effectively focuses the review on gallium and its alloys. While the melting point of gallium is 29.76 °C, alloying with other metals such as indium and tin can lower the melting point below room temperature. The composition with the lowest melting point is called the eutectic. The two most popular examples are eutectic gallium‐indium (EGaIn, 75 wt% gallium and 25 wt% indium) with a melting point of 15.7 °C and Galinstan (a eutectic typically composed of 68 wt% of gallium, 22 wt% of indium and 10 wt% of tin) with a melting point of ≈11 °C. We note that the melting point of Galinstan is often reported at −19 °C, although we could not find an original reference for this value and suspect the low value may reflect the tendency of liquid metals to supercool. Differential scanning calorimetry measurements show a melting point closer to 11 °C. Aside from differences in melting point, most gallium‐based liquid metals possess similar physical and chemical properties to a first approximation.^[^ ^42^ ^]^

Although we focus here on gallium and its alloys, we note that several alloys have melting points above room temperature (e.g., Field's metal, which melts at 62 °C) yet are metastable in the liquid state at room temperature. This feature has been used to make particles that can be processed as liquids at room temperature, yet ultimately solidify for applications such as soldering and printing.^[^ ^43^ ^]^ For more information on the behavior of metals that melt above room temperature, we point the reader to a recent review.^[^ ^44^ ^]^

**Figure 1:**
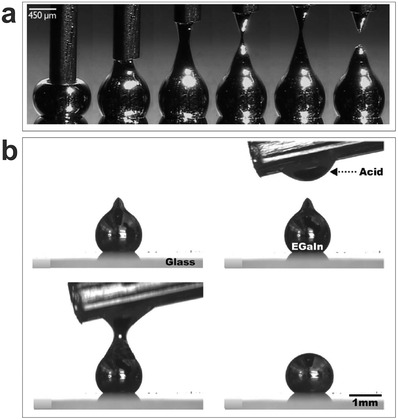
Gallium‐based liquid metals form a thin surface oxide skin. a) The oxide skin helps maintain liquid metals in non‐spherical shapes, such as the cones shown here that form by stretching a drop that spans a syringe needle and substrate. Reproduced with permission.^[^ ^56^ ^]^ Copyright 2008, Wiley‐VCH. b) A droplet can form a stable “tip” due to the oxide, yet the oxide skin can be removed by acid to change the particle shape from non‐spherical to spherical. Reproduced with permission.^[^ ^8^ ^]^ Copyright 2014, American Chemical Society.

Liquid metals based on gallium possess numerous intriguing and beneficial attributes: 1) The melting points of gallium and its alloys are near or below room temperature. In some applications, it may be appealing for metals to undergo a phase change (to absorb heat or dramatically change mechanical properties) near room temperature. Notably, liquid metals can remain in the liquid phase well below the melting point (i.e., gallium supercools).^[^ ^45^ ^]^ 2) Gallium has a negligible vapor pressure at room temperature and the vapor pressure is only ≈10^−5^ bar at 1000 °C (boiling point: ≈2400 °C).^[^ ^46^ ^]^ 3) Gallium has relatively low cytotoxicity, which makes it compatible with biological applications.^[^ ^47^, ^48^ ^]^ Gallium salts, the water‐soluble form of Ga, have even shown antibacterial properties.^[^ ^49^ ^]^ 4) These metals possess water‐like viscosity (in the absence of the oxide, the bulk viscosity of EGaIn is 1.99 × 10^−3^ Pa•s, ≈2 times that of water).^[^ ^50^ ^]^ 5) Compared to other room‐temperature liquids, such as salt water or ionic liquids, the electrical conductivity of gallium and its alloys is relatively high (See **Table** **1**).^[^ ^51^ ^]^ 6) Gallium and its alloys possess very high surface energy (≈700 mN m^−1^ for liquid gallium^[^ ^52^ ^]^ and >500 mN m^−1^ for EGaIn,^[^ ^53^ ^]^ although the exact values depend on the surrounding environment and the cleanliness of the surface). It forms a thin oxide skin spontaneously when exposed to oxygen, thereby lowering the interfacial energy of the liquid, while adding a mechanical “skin” to the surface that enables these metals to sustain non‐spherical structures (**Figure** **1**a). 7) These metals flow readily when the external pressure overcomes the yield stress of the oxide skin.^[^ ^51^ ^]^ Any freshly exposed metal reacts rapidly with air to form more oxide skin. This skin prevents the metal from flowing when the applied stress (e.g., pressure or shear) is below the surface yield stress. Thus, the oxide helps stabilize the shape of the metal. 8) The oxide skin can be removed by acid or base (Figure 1b) or via electrochemical reduction; the surface energy of EGaIn can be manipulated with less than 1 V by depositing or removing the oxide via electrochemical reactions.^[^ ^54^ ^]^ 9) Many metals can form intermetallic alloys with gallium or dissolve in gallium. It is therefore possible to change the properties of the metal, including the nature of the surface species.^[^ ^55^ ^]^

**Table 1:** Physical properties of gallium‐based liquid metals and common fluids and conductors

	Melting point [°C]	Density [g cm^−3^]	Electrical resistivity [µΩ cm]	Thermal conductivity [W m^−1^⋅K^−1^]
Gallium	29.8	5.91	27.2	30.54
EGaIn	15.5	6.25	29.4	26.43
Galinstan	10.7	6.44	30.3	25.41
DI Water	0	1	1.82 × 10^13^	0.6
Copper	1084.6	8.96	1.678	401
Silver	961.8	10.49	1.587	429
Imidazolium‐based ionic liquids (room‐temperature)	N/A (below room temperature)	1–1.4	9.524 × 10^5^–6.826 × 10^6^ (ref. [57])	N/A
PEDOT:PSS	>300^a)^	1.011	≈1 × 10^6^ (Pristine film^[^ ^58^ ^]^) as low as 217.4 (upon treatment^[^ ^59^ ^]^)	0.1–2.2 (ref. [60])

a) There is a reference indicating the melting point might be 146 °C.^[^ ^61^ ^]^

## 4 Organization of the Review

**Figure 2:**
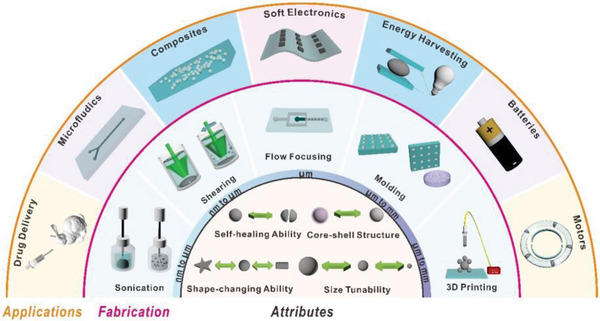
Attributes, fabrication, and applications of liquid metal particles. 1) The inner semi‐circle illustrates the attributes of liquid metal particles; 2) the middle annulus describes methods to fabricate liquid metal particles with different sizes; 3) the outer annulus presents several applications of liquid metal particles.

Inspired by the fascinating properties of liquid metals, we highlight within this review the 1) attributes of liquid metal particles; 2) methods to fabricate liquid metal particles; and 3) applications of liquid metal particles. **Figure** **2** depicts this organizational structure using three concentric semi‐circular rings. The sections along each arc depict the various attributes, fabrication methods, and applications of liquid metal particles.

### 4.1 Attributes of Liquid Metal Particles

Although gallium‐based liquid metals have a lower electrical and thermal conductivity than copper (≈one order of magnitude), they are significantly higher than conductive polymers or other room temperature conductive liquids (Table 1). In addition to having metallic electrical and thermal conductivity, liquid metal particles also exhibit several other notable features. The following section summarizes some attributes of liquid metal particles.

#### 4.1.1 Native Oxide Layer (Core–Shell Structure)

**Figure 3:**
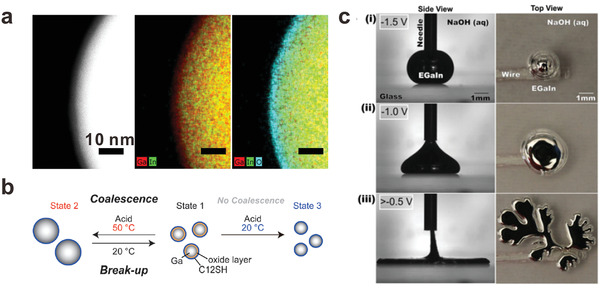
Attributes of liquid metal particles. a) TEM images of a liquid metal nanoparticle. The core is liquid EGaIn, but the solid shell (cyan) is ≈3 nm thick layer of gallium oxide. Adapted with permission.^[^ ^62^ ^]^ Copyright 2015, Wiley‐VCH. b) The size of liquid metal nanoparticles can be tuned reversibly through modulating the balance between the oxide layer and the stabilization effect of surfactants. Reproduced with permission.^[^ ^70^ ^]^ Copyright 2015, Wiley‐VCH. c) Without the oxide, the surface energy of liquid metal particles is large, yet can be lowered by applying a modest voltage that drives electrochemical oxidation of the surface. The applied voltage can lower the tension to the point that “fingers” form. Note: the open circuit potential is −1.5 V, and thus a potential of −1.5 V implies zero external voltage. Reproduced with permission.^[^ ^53^ ^]^ Copyright 2014, National Academy of Sciences.

Liquid metal particles naturally adopt a core–shell structure due to the formation of an oxide skin on the surface (vide infra). The resulting structure is similar to a water balloon since there is liquid inside a solid shell. The oxide skin is ≈0.7–3 nm thick and comprises primarily gallium oxide,^[^ ^62^, ^63^, ^64^, ^65^ ^]^ despite the fact that the gallium‐based liquid metals might contain indium or tin.^[^ ^62^, ^66^ ^]^ **Figure** **3**a shows the thickness and composition of the oxide on a particle through energy‐dispersive X‐ray spectroscopy (EDS) mapping using a transmission electron microscope (TEM). The gallium oxide on the liquid metal surface is also confirmed by other surface‐sensitive methods, such as X‐ray photoelectron spectroscopy and time‐of‐flight secondary ion mass spectrometry (ToF‐SIMS),^[^ ^64^ ^]^ angle‐resolved XPS (APXPS),^[^ ^65^ ^]^ and Auger spectroscopy,^[^ ^51^ ^]^ and it is validated through thermal oxidative composition inversion.^[^ ^67^, ^68^ ^]^ While the most stable gallium oxide structure is β − Ga_2_O_3_, the native oxide on the liquid metal surface is reported to be amorphous or poorly crystallized.^[^ ^62^, ^66^, ^69^ ^]^

There is still ongoing research to measure the oxide skin thickness and understand the oxide skin formation mechanism. Although the oxide is passivating, it does not reach its final passivating thickness immediately. For example, one study shows that it grows from ≈1.9 to ≈2.7 nm over days.^[^ ^63^ ^]^ The oxide skin growth and thickness depends on the conditions of preparation including but not limited to the oxygen concentration, water vapor concentration and surface species (e.g., thiols can bond to the metal surface and suppress its growth and surface coverage).^[^ ^63^ ^]^

#### 4.1.2 Surface Modification

Interestingly, it is possible to form other oxides on the surface of gallium by adding metals whose oxides have a more negative Gibbs free energy of formation compared to gallium oxide. For example, adding small amounts of aluminum, hafnium, or gadolinium to gallium results in the formation of a non‐native surface oxide comprising aluminum oxide, hafnium oxide, or gadolinium oxides.^[^ ^55^ ^]^ Such oxides can be separated from the oxide, which offers a route to produce oxide sheets at room temperature without the need for cleanroom‐based processing equipment.

Alternatively, thermo‐oxidation inversion can change the composition of metal alloys.^[^ ^67^, ^68^, ^71^ ^]^ For example, Galinstan naturally forms gallium oxide at room temperature but can form indium oxide and tin oxide based on thermal treatment.^[^ ^68^ ^]^ According to density functional theory (DFT) simulation, the core–shell preference largely depends on the cohesive energy and Wigner–Seitz radius (atomic size) of the metals. For instance, generally for core–shell nanoparticles formed with atoms from different groups, the metal with the largest cohesive energy goes in the core; for core–shell nanoparticles forming with atoms within a group, the metal with the smallest Wigner–Seitz goes into the core in general.^[^ ^72^ ^]^

It is also possible to modify the surface of the liquid metal particles. Molecules can anchor on the bare metal or to the gallium oxide. For example, thiols bind to metals,^[^ ^73^ ^]^ and a variety of moieties attach to oxides, such as silanes^[^ ^74^ ^]^ and phosphates.^[^ ^75^ ^]^ Initiators can be grafted to particles to grow polymer from the surface.^[^ ^76^ ^]^ Recent work shows it is possible to use gallium to initiate free radical polymerization without a traditional molecular initiator.^[^ ^39^ ^]^ Polymerization initiates by breaking the oxide layer (e.g., by sonication), thereby exposing reactive gallium to a surrounding solution of monomer. This provides a simple route to graft monomers directly to the metal. Particles can also be added to the surface of the metal to form “liquid marbles,”^[^ ^77^ ^]^ as discussed in the Section 4.3. The capability to engineer the surface properties of liquid metal particles could potentially find use in catalysis, colloidal self‐assembly, or biomedical applications.

#### 4.1.3 Sintering

Bringing two or more liquid metal particles together with sufficient stress can cause the oxide to break, thus allowing the individual particles to merge (or partially merge). The ability to sinter particles allows for the creation of conductive paths approximately equal to the length occupied by the particles prior to merging. Whereas solid metallic particles only sinter (i.e., form conductive contacts) using high temperatures or intense flashes of light, liquid metal particles can be mechanically sintered at room temperature and result in soft, stretchable conductors.^[^ ^62^, ^78^ ^]^ The stress needed to rupture the particles can be tuned based on the size of the particles or by changing the coating on the liquid metal.

#### 4.1.4 Self‐Healing via Merging

The ability to merge liquid metal particles endows devices with self‐healing properties that are useful for conductive traces in electronics.^[^ ^78^, ^79^, ^80^, ^81^, ^82^, ^83^ ^]^ In addition to particles merging, liquid metals can also “smear” in a useful way. Cutting a composite containing liquid metal particles can cause them to smear along the walls of the cut to create a conductive path, thereby allowing liquid metal conductors within soft, conductive composites to retain conductivity after being damaged.^[^ ^83^ ^]^ In addition to inducing healing mechanically, self‐healing has also been demonstrated using chemical processes that use etchants to create point contacts between particles.^[^ ^84^ ^]^

#### 4.1.5 Size Tunability

While it is generally possible to synthesize/fabricate metallic particles of various sizes, it is challenging to change their sizes afterward. However, liquid metal particles can be physically broken into ones with smaller sizes (e.g., by sonication^[^ ^85^ ^]^), and likewise, particles can merge by bringing them in contact with sufficient force, as mentioned previously.^[^ ^62^, ^78^ ^]^ The balance between the break‐up and coalescence of a suspension of liquid metal nanoparticles can also be adjusted by varying the pH to modulate the stabilizing effects of the oxide (Figure 3b).^[^ ^70^ ^]^

#### 4.1.6 Giant Tunability of Interfacial Tension

The interfacial tension of liquid metal can be tuned reversibly over an unprecedented range using electrochemical reactions requiring less than 1 V applied to the metal relative to a counter electrode.^[^ ^53^ ^]^ In the absence of oxide, the metal has a large interfacial tension (>500 mN m^−1^) Yet, electrochemical deposition of the oxide causes a precipitous drop in tension. In NaOH solution, the oxide dissolves during deposition; thus, the metal can flow despite the presence of oxide species on the surface. As the interfacial tension lowers, a droplet of metal just below the capillary length converts from a spherical shape to a flat shape due to gravity. With increased potential (≈0.8 V applied potential), a droplet of the metal expands significantly while forming a fractal structure, suggesting the interfacial tension approaches zero (Figure 3c). The potential serve as a nice strategy to control the interfacial tension to manipulate liquid metals.^[^ ^54^ ^]^ Within a confined geometry, a liquid metal droplet can spread (due to gravity) to fit the corresponding shape of the container, yet, in the absence of voltage, the NaOH dissolves the oxide and the metal reverts back to a spherical shape due to the large surface energy of the bare metal.^[^ ^53^, ^54^, ^86^ ^]^ We emphasize that the ability to tune the tension is due to surface oxidation, and not classic electrocapillarity.^[^ ^54^ ^]^

#### 4.1.7 Shape‐Changing Ability

In addition to using potential to control the effective tension of liquid metal, there are other ways to change the shape in response to a stimulus. Liquid metal nanoparticles can transform from spherical liquids to cylindrical GaOOH rods upon heating.^[^ ^87^, ^88^ ^]^ The heat can also be delivered using light absorbed by graphene‐oxide species adhered to the surface of liquid metal particles. Liquid metal particles can also change diameter by merging under controlled conditions, as shown in Figure 3b.

### 4.2 Fabrication of Liquid Metal Particles

In general, metallic particles can be produced in many ways, including chemical reduction of metallic salts,^[^ ^89^ ^]^ microemulsions,^[^ ^90^ ^]^ chemical vapor deposition,^[^ ^91^ ^]^ microwave irradiation,^[^ ^92^ ^]^ and others. Perhaps one of the easiest ways to create nanoparticles is to reduce metallic salt precursors. This was the approach developed by Michael Faraday to create gold nanoparticles in the 1850s.^[^ ^89^ ^]^ It is very difficult to reduce gallium salt precursors into gallium nanoparticles since gallium serves as a strong reducing agent. A recent report manages to use gallium alkylamides as precursors and synthesizes gallium nanoparticles through the judicious choice of the reaction parameters.^[^ ^93^ ^]^ Alternatively, gallium micro/nanoparticles can be deposited on substrates via thermal deposition or by molecular‐beam epitaxy, although these techniques require specialized equipment and provide poor control over the geometry of the particles.^[^ ^94^, ^95^, ^96^, ^97^ ^]^ Here, we will discuss ways to fabricate liquid metal particles with a variety of sizes using top‐down approaches that are easy to implement due to the fluid nature of liquid metals.

#### 4.2.1 3D Printing and Drop‐on‐Demand Dispensing (Tens of µm to a Few mm)

**Figure 4:**
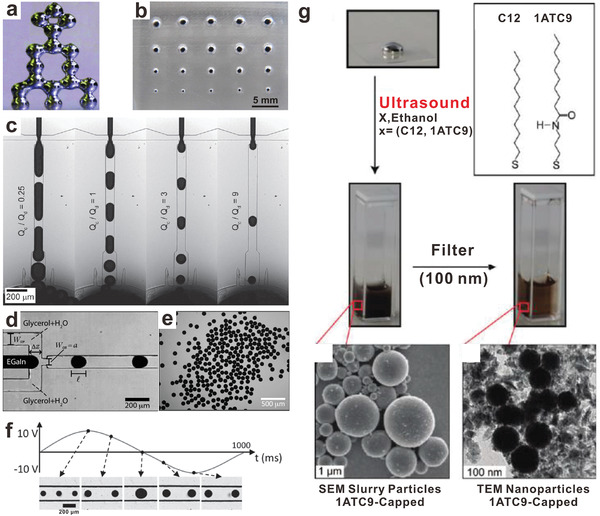
Liquid metal particles produced with a variety of fabrication methods. a) A 3D “tower” stacked with liquid metal particles produced by 3D printing. Reproduced with permission.^[^ ^98^ ^]^ Copyright 2013, Wiley‐VCH. b) Molding to produce liquid metal particles with various sizes. Reproduced with permission.^[^ ^102^ ^]^ Copyright 2014, MDPI. c) Microfluidic flow‐focusing to produce liquid metal particles. Changing the flow rate ratio controls the droplet size. Note: *Q*_C_is the continuous phase flow rate and *Q*_d_ is the liquid metal flow rate. Reproduced with permission.^[^ ^103^ ^]^ Copyright 2012, Wiley‐VCH. d,e) A flow‐focusing device to produce homogenous liquid metal microparticles. Reproduced with permission.^[^ ^104^ ^]^ Copyright 2012, Royal Society of Chemistry. f) The size of liquid metal microparticles can be tuned with modest voltage (−10 to 10 V) in flow‐focusing devices. Reproduced with permission.^[^ ^105^ ^]^ Copyright 2015, Wiley‐VCH. g) Fabrication of liquid metal micro/nanoparticles via ultrasound, followed by filtering to remove larger particles. Adapted with permission.^[^ ^85^ ^]^ Copyright 2011, American Chemical Society.

The fluidic nature of liquid metals makes them compatible with numerous patterning techniques. Perhaps the easiest approach to produce liquid metal particles is through manual syringe/pipet extrusion.^[^ ^98^ ^]^ Arbitrary 3D conductive structures can be produced through stacking the liquid metal particles or chaining them together (**Figure** **4**a).^[^ ^99^, ^100^, ^101^ ^]^ During the printing process, the applied pressure overcomes the yield stress of the oxide skin to dispense the metal as spherical particles. The newly formed oxide skin helps to stabilize the structures afterward. In our experience, droplets produced this way are, at minimum, tens of microns in size, but typically hundreds of microns in diameter depending on the size of the nozzle and the temporal pressure profile that forces the metal from the nozzle. Note that forcing the metal from a smaller nozzle requires a larger pressure, but as the metal exits the nozzle, the pressure must immediately decrease to avoid expanding the droplet to larger geometries. It is thus hard to create small droplets in this manner.

It is extremely difficult to directly inkjet print liquid metal due to the extraordinarily high surface tension (and/or the native oxide), which makes it challenging to push the metal from small orifices. Yet, dispersing liquid metal nanoparticles in suitable solvents provides a potential way to inkjet print liquid metal from a suspension to prepare various flexible/soft electronics with high resolution.^[^ ^78^ ^]^ Such printed particles can be rendered into a conductive trace by mechanical sintering.

#### 4.2.2 Molding (Tens of µm to a Few mm)

Molding generally refers to a fabrication process that involves shaping liquid or pliable raw materials using a mold. It is possible to mold liquid metal to produce particles.^[^ ^102^ ^]^ In this molding procedure, liquid metal is pressed into a mold with recessed features. Removing the oxide skin with acid vapor releases the particles. The size of thus‐obtained liquid metal particles correlates with the volume of the reservoir (Figure 4b). In this specific example, the molds produce particles ranging from hundreds of microns to several millimeters. Based on efforts to mold lines or traces of liquid metal, it should be possible to create structures as small as 1 µm through the use of molds with smaller features^[^ ^106^ ^]^ or selective wetting.^[^ ^33^ ^]^ However, this has not yet been demonstrated for particles. The ability to fill the mold with metal limits the smallest particles that can be created by molding. In principle, if the metal wets the mold (e.g., by using a thin metal coating on the surface of the mold), the necessary pressure should decrease, yet it then becomes challenging to remove the metal from the mold if it naturally wets the metal coating. Molding has the appeal of being simple while providing control over the size (and size distribution) of the particles. In addition, the molds organize the particles spatially. Therefore, it is possible to subsequently transfer arrays of organized particles to other surfaces. Although, in principle, molding can be scaled to be a high throughput technique, in a laboratory environment, this method produces fewer particles in a given amount of time relative to other techniques.

#### 4.2.3 Microfluidic Production (50–200 µm)

Microfluidics offers a convenient way for fabricating droplets in a controlled and reproducible manner. This approach can produce particles with a narrow distribution of diameters with ≈1–3% in polydispersity (defined as the standard deviation of the size distribution divided by the mean droplet size).^[^ ^107^ ^]^ In microfluidic production, liquid metal and an immiscible “continuous” phase are pumped simultaneously into a microchannel. The shear forces that arise as these two fluids pass through a constriction cause the metal to deform into droplets. The droplet diameter reflects the competition between shear and interfacial forces (the former favors smaller particles, and the latter favors larger particles). Multiple studies^[^ ^103^, ^104^, ^108^, ^109^, ^110^, ^111^, ^112^ ^]^ have demonstrated the possibility to form uniform liquid metal microparticles through microfluidic production (Figure 4c–e). Often, the fluids are pumped through an orifice in a so‐called “flow‐focusing” microfluidic device. The continuous phase could be, for example, an aqueous solution (i.e., mixture of glycerol and water or polyethylene glycol‐electrolyte solution) or oil (i.e., silicone oil). The continuous phase needs to be sufficiently viscous to shear the metal into particles as they pass through the orifice. The oxide that forms on the particles helps stabilize the particle size, and the addition of surfactants (e.g., polyvinyl alcohol) prevents them from coalescing. After the formation of the liquid metal microparticles, it is also possible to hydrodynamically transfer the particles across a fluid–fluid interface into other fluidics within a monolithic chip.^[^ ^110^ ^]^ Interestingly, microfluidic fabrication can also produce oval particles^[^ ^103^ ^]^ or even liquid metal fibers.^[^ ^105^ ^]^

Microdroplets manufactured in this way are typically tens to hundreds of microns in diameter; the size can be tuned by adjusting the flow rates, microfluidic channel geometry, the viscosity of the continuous phase, and the interfacial energy between the two phases, similar to other fluidic systems.^[^ ^113^ ^]^ In principle, it may be possible to create smaller size liquid metal particles by increasing the shear rate, but the high pressures required to do so can cause the microfluidic devices to delaminate.

One of the exciting aspects of using liquid metals is that electrical potential (Figure 3c) can tune the surface energy of liquid metal in real time, as shown in Figure 4f. There are two ways to use potential to lower the tension: electrical double layers and surface anodization (electrochemically driven oxidation). The electrical double layer that forms at the interface lowers the surface energy, as described by Lippmann's equation:^[^ ^105^, ^114^ ^]^

(1) $$\gamma =\gamma_{0}-\frac{1}{2}C_{\text{EDL}}V_{\text{EDL}}^{2}$$

where γ is the interfacial tension, γ_0_ is the maximum interfacial tension when the electrical potential across the electrical double layer (EDL) *V*_EDL_ is 0 V. *C*_EDL_is the capacitance of the electrical double layer. With the increase of voltage across the electrical double layer, *V*_EDL_, the interfacial tension γ decreases and the droplets decrease in size.

In addition, electrochemical oxidation of the surface can occur at higher potentials and further lower the surface energy.^[^ ^53^, ^54^ ^]^ As such, the diameter of the liquid metal microparticles can be tailored in real time, resulting in microspheres, some of which have diameters even 25% smaller than the width of the orifice (which is atypical in flow focusing).^[^ ^105^ ^]^ In addition, the use of alternating potentials (e.g., square waves) can create unique and well‐controlled distributions of particles (e.g., a near‐perfect bimodal distribution of diameters or other controlled particle distributions).

#### 4.2.4 Ultrasonication (Tens of nm to a Few µm)

Ultrasound, through acoustic cavitation, causes the formation, growth, and implosive collapse of bubbles in a liquid, in which localized hot spots have very high temperatures (≈5000 °C) and very high pressures (≈500 atmospheres). Ultrasound is a powerful and facile method to produce liquid metal micro/nanoparticles.^[^ ^115^ ^]^ In a typical experiment, liquid metal is added into a vial filled with liquid (e.g., ethanol) and placed in an ultrasonic bath for a few hours, followed by filtering or centrifugation to remove larger particles. The addition of molecules such as 1‐dodecanthiol/3‐mercapto‐*N*‐nonylpropionamide leads to the formation of self‐assembled monolayers (SAMs) on the surface of the particles and hence stabilizes them and minimizes coalescence (Figure 4g).^[^ ^85^ ^]^ One systematic study investigated the stabilization effect of thiols with different alkyl chains and revealed that 1‐octadecanethiol was the most efficient thiol tested for binding to the metal for particle stabilization.^[^ ^116^ ^]^ Phosphonic acid functionalization is effective as well.^[^ ^117^ ^]^ Interestingly, liquid metal nanoparticles in ethanol (and oxygen) avoid precipitation for a few weeks even without any SAM layers attached to the particle surface. In contrast, other liquids, such as acetone, dodecane, methanol, produce particles that are not stable over the same time frame despite the presence of an oxide layer on the particles. The stability of the particles formed in ethanol is most likely due to a carbon layer coating on the surface of the nanoparticles,^[^ ^62^, ^66^ ^]^ although the exact reason for the formation of this layer is poorly understood.

Generally, liquid metal nanoparticles in aqueous solution are not stable since gallium tends to react with water and oxygen to form gallium oxide monohydroxide, and the suspension will precipitate (in our experience, it precipitates within one hour at room temperature). Yet, it is possible to stabilize the colloidal liquid metal nanoparticles in aqueous solution in the presence of certain surfactants^[^ ^87^ ^]^ or a hydrophilic polymer^[^ ^118^, ^119^ ^]^ for a few days. It is also possible to manipulate the size of the nanoparticles during sonication (at least to some extent). Under ultrasonication, the balance between the break‐up and coalescence of the gallium nanoparticles can be adjusted by changing the temperature or adding acid, which removes the native surface oxide layer and adding thiols that adsorb to the exposed metal surface and stabilize the particles.^[^ ^70^ ^]^ Gallium alloy nanoparticles can also transform into solid Janus nanoparticles after temperature cycling due to phase separation of gallium and other metals (e.g., indium).^[^ ^120^ ^]^

While an ultrasonic bath is easily accessible in most laboratories and provides sufficient energy to break the liquid metal into particles into smaller units, probe sonication reduces the processing time to a few minutes.^[^ ^62^, ^66^, ^70^, ^78^, ^121^ ^]^ On‐chip sonication can create acoustic waves that break liquid metal into micro‐ to nano‐particles. It can also control the size of the particles by tuning the interfacial energy via electrochemistry or electrocapillarity.^[^ ^119^, ^122^ ^]^

#### 4.2.5 Shearing (a Few nm to a Few µm)

Shearing is a straightforward method to break a volume of liquid metal into particles. In a typical shearing experiment, liquid metal is placed in solution, for example, acetic acid, and sheared using a stirring apparatus. Initially, shearing elongates the liquid metal locally to a cylindrical shape that breaks into droplets upon reaching the Rayleigh–Plateau limit in length. The method is termed SLICE (shearing liquids into complex particles).^[^ ^43^ ^]^ SLICE can form a wide range of sizes by changing the shearing speed and the shearing liquid. Particles range from a few nm to a few µm. Since this shearing process involves air and water, the generated liquid metal will possess an oxide skin, similar to the ultrasound process. The shearing method is applicable for other low‐melting alloys, such as Field's metal.^[^ ^84^, ^123^ ^]^ The resulting particles can be applied for ambient soldering,^[^ ^123^ ^]^ electrical interconnect fabrication,^[^ ^84^ ^]^ and self‐stiffening materials.^[^ ^124^ ^]^

### 4.3 Applications of Liquid Metal Particles

#### 4.3.1 Drug Delivery

Drug delivery involves transporting a pharmaceutical compound into the body to safely achieve a desired therapeutic effect. Generally, direct delivery of drugs or biomolecules is not efficient as it suffers from numerous shortcomings. Therefore, it is desired to explore safe and efficient carriers for drug delivery, among which inorganic nanoparticles are promising candidates.

Given the toxicity of mercury, “liquid metals” may be generally assumed to be toxic. Yet, gallium‐based liquid metals possess very low toxicity and are suitable candidates as carriers for drug delivery.^[^ ^47^, ^88^, ^125^, ^126^, ^127^, ^128^, ^129^, ^130^ ^]^ A recent review highlights some biomedical applications of liquid metals,^[^ ^131^ ^]^ and here we will only discuss liquid metal nanoparticles.

**Figure 5:**
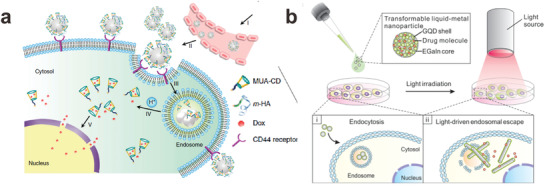
Liquid metal nanoparticles for drug delivery. a) Nanomedicine based on liquid metal nanoparticles can enter tumor cells by endocytosis and fuse under low pH to accelerate the release of drugs from the surface of the particles. MUA‐CD is a thiolated cyclodextrin molecule that binds to themetal particles and holds doxorubicin (Dox), a cancer drug. m‐HA is a thiolated hydraluronic acid that binds to the metal particle surface and serves as a targeting moiety for the CD44 receptor, which is over‐expressed on tumors. Reproduced with permission.^[^ ^47^ ^]^ Copyright 2015, Nature Publishing Group. b) Coated with graphene quantum dots, liquid metal nanomedicine can transform from a spherical shape to a solid rod shape in tumor cells when exposed to near‐IR light due to the oxidation of the metal. Reproduced with permission.^[^ ^88^ ^]^ Copyright 2017, American Chemical Society.

As one example, liquid metal nanoparticles can be prepared via sonication in the presence of both drugs (e.g., doxorubicin) and functional groups for tumor recognition that bind to the surface of the particles. The as‐prepared particles target tumor cells and enter them by endocytosis. Interestingly, the prepared nanomedicine is pH‐responsive; namely, the oxide skin on the liquid metal nanoparticles can be removed inside the acidic tumor region. Thus, drugs loaded on the particles release and the liquid metal nanoparticles fuse together to accelerate the drug release in tumor regions, followed by degradation of liquid metal (**Figure** **5**a). The fused nanoparticles display a contrast‐enhancing capability imaged by X‐ray, suggesting their potential as theranostic agents. This type of nanomedicine shows excellent efficiency in both tumor targeting and antitumor performance. Importantly, a systematic investigation has demonstrated that liquid‐metal‐based nanomedicine shows no obvious toxicity at the applied treatment dose in mice; in vivo metabolism study indicates that the clearance of nanomedicine is possible through both fecal and renal excretions.^[^ ^47^ ^]^

A recent study demonstrates that liquid metal shows relatively low cytotoxicity, although the release of indium during sonication has some toxicity (notably, since the temperature of the body is above the melting point of gallium, indium is not necessary for in vivo applications of liquid metals).^[^ ^132^ ^]^ Gallium salts have been FDA approved for several pharmaceutical and imaging applications. While the aforementioned studies demonstrate the low toxicity of gallium‐based liquid metals, additional studies are needed to better comprehend the impact of liquid metals on humans.

In addition to delivering surface‐mounted drugs, gallium‐based nanoparticles can shape transform as a route to disrupting cells. In the absence of appropriate stabilizing ligands, liquid metal nanosphere can transform into gallium oxide hydroxide nanorods in the presence of water due to the oxidization of gallium.^[^ ^87^ ^]^ If this transformation happens quickly and selectively, it can be harnessed to disrupt cells. Near‐IR light is attractive as a stimulus to induce this shape change because human skin is partially transparent to this wavelength of light. Gallium‐based nanomedicines become responsive to near‐IR light upon appropriate surface modification. Coated with graphene quantum dots, the spherical liquid metal nanoparticles absorb IR light, which drives the shape‐changing process. Within tumor cells, the shape transformation from nanospheres to nanorods not only accelerates drug release but also breaks the endosomal membrane to achieve more efficient cancer treatment (Figure 5b) In addition, reactive oxygen species generated by the IR light/laser radiation also effectively eliminate cancer cells.^[^ ^88^, ^125^, ^126^, ^127^, ^128^, ^129^, ^130^, ^133^, ^134^ ^]^

Liquid metal particles can also be manipulated to undergo locomotion. Interestingly, with a nickel cap on a millimeter‐scale liquid metal droplet, the resulting particle can behave as motors that can be manipulated under ansi electrical field and magnetic field. When loaded with alginate‐based biomaterials containing aluminum nanoparticles as drugs, such a motor exhibits steerable motion for drug delivery in a proof‐of‐concept experiment.^[^ ^135^ ^]^

#### 4.3.2 Microfluidics

A recent review highlights the advantages and applications of liquid metals for microfluidics.^[^ ^136^ ^]^ Here, we focus on microfluidics that use droplets of liquid metals. By precisely controlling the location and volume of the droplets within the microchannels, it is possible to use liquid metal for a variety of tools.^[^ ^137^ ^]^

##### Pump

**Figure 6:**
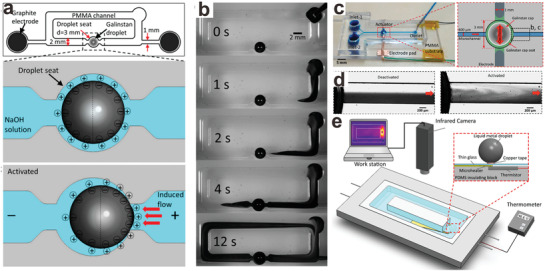
Microfluidics that utilize liquid metal particles. a) Schematic of the liquid metal enabled pump and its pumping mechanism. b) Screenshots of the pumping process of a fluid dyed black. The scale bar is 2 mm. Adapted with permission.^[^ ^138^ ^]^ Copyright 2014, National Academy of Sciences. c) Schematic of a liquid metal‐enable mixer. The scale bar is 5 mm. d) Screenshot of the two immiscible liquid phases when the mixer is deactivated/activated. The scale bar is 200 µm. Adapted with permission.^[^ ^138^ ^]^ e) Schematic of the cooling system enabled by liquid metal particles. Reproduced with permission.^[^ ^139^ ^]^ Copyright 2016, American Chemical Society.

In microfluidics, pumps are essential to driving liquid flow in the channels, and liquid metal particles can be employed as pumps. As shown in **Figure** **6**a, when voltage is applied to the metal in an electrolyte, the charges at the interface will repel each other, thereby decreasing the interfacial energy (according to Lippmann's equation for electrocapillarity, Equation (1)).^[^ ^138^ ^]^ When a charge gradient exists on the particle surface, a gradient in tension forms and Marangoni flow occurs. Since the metal rests in a “seat” and cannot move, the electrolyte moves across the surface of the metal sphere, resulting in pumping (Figure 6b).^[^ ^138^ ^]^

##### Mixer

Similar to the pumping mechanism, liquid metal particles in solution under a sinusoidal signal induce harmonic Marangoni flow, thus generating vortices within the solution. Such a phenomenon can be integrated into a microchannel system to induce chaotic advection to achieve highly efficient mixing (Figure 6c,d).^[^ ^140^ ^]^

##### Cooler

Pumping can be used for thermal cooling. Liquid metal particles placed over a hot spot within a microfluidic system can serve as a micropump to remove heat convectively. In addition to pumping the surrounding fluid, the high thermal conductivity of liquid metal can also help to dissipate heat (Figure 6e).^[^ ^139^ ^]^

#### 4.3.3 Composites

Polymer–metal composites that contain liquid metal particles dispersed in elastomer are attractive due to their ability to tune the electrical, thermal, and mechanical properties of the elastomer without stiffening the composite as occurs with solid fillers. Interestingly, at relatively low volume loading, similar properties could be achieved as solid fillers.^[^ ^141^ ^]^ Recent reviews highlight the advantages and the recent progress of liquid metal and polymer composites.^[^ ^142^ ^]^ Thus, we only briefly discuss them here.

**Figure 7:**
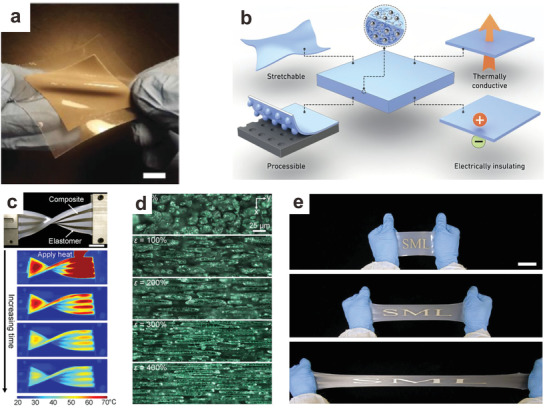
Composites of liquid metal particles dispersed within elastomer. a) Photograph of a soft, flexible, and stretchable liquid metal elastomer composite. Scale bar is 10 mm. Adapted with permission.^[^ ^143^ ^]^ Copyright 2016, Wiley‐VCH. b) These composites are stretchable, easy to process and provide enhanced thermal conductivity yet often remain electrically insulating. Reproduced with permission.^[^ ^148^ ^]^ Copyright 2015, Nature Publishing Group. c) Flexible liquid metal elastomer composite can dissipate heat quickly, demonstrating a high thermal conductivity. Scale bar is 25 mm. Adapted with permission.^[^ ^149^ ^]^ Copyright 2017, National Academy of Sciences. d) Morphology of liquid metal elastomer under 0%, 100%, 200%, 300%, and 400% strain illustrates how the liquid particles align with strain and thereby affect thermal conductivity. The images are taken under an optical microscope. Scale bar is 25 µm. Adapted with permission.^[^ ^149^ ^]^ Copyright 2017, National Academy of Sciences. e) Highly stretchable liquid metal elastomer composite with excellent dielectric properties Scale bar is 5 cm. Reproduced with permission.^[^ ^150^ ^]^ Copyright 2016, Wiley‐VCH.

Soft composites can be manufactured by dispersing liquid metal into elastomer matrices. To produce liquid metal‐elastomer composites, it is possible to break the liquid metal into particles in situ by mixing liquid metal with elastomer pre‐polymer. Alternatively, it is possible to prepare the particles first, followed by mixing with an elastomer precursor prior to curing the composite. One of the first reports of liquid metal‐elastomer composites mixed liquid metal with uncured polydimethylsiloxane (PDMS) via a mortar and pestle^[^ ^143^ ^]^ (**Figure** **7**a). Under shear, the liquid metal breaks into smaller particles and subsequent curing “cages” the particles within the PDMS network. As made, the composite was non‐conductive. However, it turned into a conductive system by applying a localized compressive force that tears or ruptures the elastomer walls surrounding the liquid metal particles to form a continuous network of liquid metal. The composite can be stretched to >100% strain repeatedly while retaining relatively high conductivity (1.05 × 10^4^ S m^−1^). Such composites with excellent electrical properties could be engineered as sensors for damage detection or soft robotics with self‐healing properties.^[^ ^144^ ^]^ Peeling of these composites from a surface also generates enough force to cause particles to merge.^[^ ^145^ ^]^ The mechanical and electrical properties of such composites or their pre‐cured dispersions were systematically studied by rheology and by X‐ray computed tomography. These studies show that decreasing the size of the liquid metal will increase the viscosity of the dispersion.^[^ ^146^ ^]^ The size and loading of particles also affects the properties of the resulting liquid metal soft composite. These liquid metal based composites can be used as wearable sensors to monitor the movement of a finger due to the capacitance change during geometric changes of the conductor.^[^ ^147^ ^]^

Composites consisting of liquid metal particles within elastomer possess excellent thermal properties relative to other soft materials.^[^ ^76^, ^148^, ^149^ ^]^ The thermal conductivity of such composites increases significantly with increased loading of liquid metal particles (Figure 7b). Under stress, the soft liquid metal droplets in the elastomer network deform and the morphology of the composite changes to create pathways with improved thermal conductivity in the direction of strain relative to the composite at rest.^[^ ^149^ ^]^ (Figure 7c,d). The unique combination of high thermal conductivity and elasticity is promising for creating soft devices with rapid heat dissipation.

Liquid metal elastomer composites also feature enhanced dielectric properties. Dielectric elastomers are used for Maxwell stress‐induced actuation due to their relatively low Young's modulus. Such systems are promising for generating soft sensors or for manufacturing soft robotic systems.^[^ ^151^ ^]^ However, most dielectric elastomers possess low dielectric constants. Materials with low dielectric constants require very high electric fields to cause actuation. Generally, adding inorganic fillers, such as metallic powders, carbon materials, or ceramic materials, can increase the dielectric properties. Yet, this method suffers from a concurrent dramatic increase of modulus of the composite, which compromises the actuation behavior. In contrast, the dielectric performance of liquid metal‐elastomer composites can improve without noticeably increasing the composite modulus. Integrating liquid metal microparticles into silicone elastomers can increase the dielectric constant by over 400% (relative to elastomer without particles) with a low dielectric dissipation factor while allowing the composite to be stretched multiple times its original length (Figure 7e).^[^ ^150^ ^]^ Depending on the size of the liquid metal particles and the loading volume percentage, the Weibull breakdown strength (dielectric breakdown) could be in the range of 1 to 100 kV mm^−1^. Notably, adding liquid metal particles can even toughen the elastomer dramatically by increasing energy dissipation and redirecting crack propagation normal to the direction of the crack. This effectively eliminates the crack tip where stress normally concentrates, making it an ultra‐tough but soft material.^[^ ^152^ ^]^ Recent work functionalized liquid metal nanodroplets with a surface‐initiated atom transfer radical polymerization to form polymeric ligand encapsulation to stabilize liquid metal nanodroplets.^[^ ^76^ ^]^ Compared to mechanical mixing, such method could render more regular‐shaped particles to reduce the anisotropic distribution of composite properties.^[^ ^76^ ^]^

Soft composites containing liquid metal could also incorporate other fillers to achieve new functionality. For instance, liquid metal‐filled magnetorheological elastomer comprising a hybrid of fillers of liquid metal microdroplets and metallic magnetic microparticles exhibits an unconventional positive piezoconductive effect. Normally, elastomeric composites containing conductive particles become more resistive (piezoresistive) when deformed because the deformation increases the distance between the particles. Liquid metals, however, can form soft and deformable contacts between rigid particles and therefore undergo piezoconductivity; that is, the conductivity of the composite increases by orders of magnitude under compression or tension.^[^ ^153^ ^]^

#### 4.3.4 Soft Electronics

It is possible to create flexible electronics using rigid conductors (e.g., Cu or Au) by simply making the conductive traces thin. These same materials can be rendered stretchable by patterning them into strategic geometries (e.g., meander serpentine patterns) or by embedding nanoparticles of rigid electrical conductors into the elastomer.^[^ ^1^, ^154^, ^155^ ^]^ Although thin conductors can conform readily to surfaces with minimal force (and thus, feel “soft”), truly soft electronics require the use of intrinsically soft materials. The fluidic and metallic nature of liquid metal makes liquid metals great candidates for soft electronics. A recent review highlights the progress in liquid metal‐based stretchable electronics.^[^ ^2^ ^]^ Soft electronics applications involving liquid metal particles will be discussed within several categories outlined below.

##### Soft Conductors

**Figure 8:**
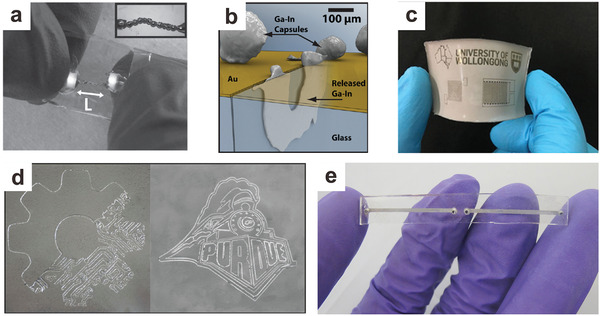
Soft electronics based on liquid metal particles. a) Stretchable interconnects using 3D printing liquid metal droplets (length, *L*, is ≈1 cm). Adapted with permission.^[^ ^98^ ^]^ Copyright 2013, Wiley‐VCH. b) Self‐healing circuits with liquid metal particle capsules. Upon mechanical damage, the capsules will break to release the inner liquid metal to restore conductivity. Adapted with permission.^[^ ^159^ ^]^ Copyright 2011, Wiley‐VCH. c) A soft circuit based on liquid metal nanoparticles (the device has diameters of approximately several cm in width and length). Adapted with permission.^[^ ^66^ ^]^ Copyright 2016, Wiley‐VCH. d) Spray printing of liquid metal particles followed by mechanical sintering into conducive patterns (the image is approximately several cm in width and length). Adapted with permission.^[^ ^160^ ^]^ Copyright 2017, Wiley‐VCH. e) A soft antenna device based on microfluidics and liquid metal nanoparticles. The conductive length of the antennas can be tuned via mechanical sintering to alter the resonant frequency. Adapted with permission.^[^ ^62^ ^]^ Copyright 2015, Wiley‐VCH.

Liquid metals are intrinsically suitable to produce soft conductors due to their metallic and soft nature. Liquid metal particles produced by 3D printing^[^ ^98^, ^156^ ^]^ or inkjet printing^[^ ^157^ ^]^ can be used for stretchable interconnects to electrically connect circuit elements (**Figure** **8**a). It is also possible to draw conductive liquid metal wires (as small as a few microns) at room temperature via stretching a liquid metal particle on a viscoelastic polymer substrate.^[^ ^158^ ^]^ There are many other ways to make liquid metal patterns, but we limit the discussion here to only particle‐based strategies.

##### Soft Circuits

Liquid metal particles can be integrated into circuits and endow them not only with stretchability, but also with self‐healing and responsive characteristics.

1) *Self‐Healing Circuits*. Liquid metal particles encapsulated within polymer shells can be integrated over gold circuit traces. When the circuit is cut or broken, and the gold trace loses electrical continuity, the liquid metal will rupture from its encasement and reconnect the conductive path, thus restoring the functionality of the circuit.^[^ ^81^, ^159^ ^]^ (Figure 8b). The mechanical properties of the polymer shell can be engineered to tune the force to trigger the self‐healing performance.^[^ ^63^ ^]^ Liquid metal microcapsules can also be utilized as self‐healing conductors for sustainable and flexible perovskite solar cells.^[^ ^161^ ^]^
2) *Pressure‐Responsive*. Nanoparticles can be cast as thin films (micron thick) and embedded between two PDMS pads. This “sandwich” structure can be employed as a soft circuit board with different circuit components. Initially, the film is not conductive. Yet, under located mechanical stress, the oxide skin on the particles will rupture, and the liquid metal will merge to form a conductive path, akin to rupturing small water balloons (Figure 8c) via “mechanical sintering.”^[^ ^62^, ^66^, ^78^ ^]^ While conventional sintering requires high temperatures to merge particles together, the “mechanical sintering” approach can be performed at room temperature.^[^ ^78^ ^]^ Liquid metal elastomer composites can achieve similar results but with lower electrical conductivity than the abovementioned “sandwich” structure.^[^ ^143^ ^]^ It is possible to use a non‐contact method, that is, laser sintering, to connect the liquid metal particles, thus achieving the transition from a non‐conductive to a conductive pathway with relatively high‐resolution conductive patterns.^[^ ^62^, ^71^, ^162^ ^]^ Alternatively, liquid metal particle inks can be prepared and spray‐printing to fabricate different devices, followed by mechanical sintering into conductive patterns (Figure 8d).^[^ ^160^ ^]^
3) *Soft Antennas*. Liquid metal is a suitable candidate for soft and stretchable antennas due to its metallic electrical conductivity, which is necessary for an antenna to radiate efficiently. A variety of soft antennas have been designed and fabricated using liquid metals, and the antennas can tune their spectral properties accordingly by changing the shape of the liquid metal my deforming the matrix that hosts the liquid metal guest.^[^ ^20^, ^21^, ^22^, ^23^, ^24^, ^25^, ^26^, ^28^, ^163^ ^]^ In addition, the ability to mechanically sinter liquid metal nanoparticles to create conductive trances has provided the ability to tune the resonant frequency of the antenna by altering the length of the conductive path (Figure 8e).^[^ ^62^ ^]^

#### 4.3.5 Energy Harvesting

The rapid consumption of nonrenewable oil resources motivates humans to identify alternative ways to harvest energy. Energy harvesting based on environmental and mechanical energy, that is., vibration and human movement, is promising, especially for wearable devices. In this regard, reverse electrowetting provides a potential way to convert mechanical to electrical energy.^[^ ^164^ ^]^ In a classic electrowetting experiment, the wettability of conductive liquid particles on a dielectric surface increase with electrostatic energy (through applying a potential between the droplets and the dielectric‐coated electrode). Electrowetting effectively converts electrical energy to mechanical energy (particle movement). In reverse electrowetting, mechanical manipulation of the liquid particles varies the geometry of a capacitor and thereby moves charge through a circuit. Liquid metals represent an ideal conductive fluid for reversible electrowetting due to their high electrical conductivity, high surface energy, and low vapor pressure compared to other conductive fluidics. To date, experiments on reverse electrowetting focus on mercury due to the tendency of gallium‐based liquids to adhere to surfaces.^[^ ^164^ ^]^ A variety of techniques have emerged to prevent adhesion, including using slip layer (thin liquid layers between the oxide and substrate),^[^ ^165^ ^]^ rough surfaces,^[^ ^166^, ^167^, ^168^, ^169^, ^170^ ^]^ and acidified oils.^[^ ^171^ ^]^

#### 4.3.6 Switches, Sensors, and Transistors

Liquid metal electrowetting can be applied for droplet‐based microswitches. Droplet‐based microswitches using mercury have been used widely over the past few decades.^[^ ^172^, ^173^ ^]^ There are a few drawbacks using gallium‐based liquid metal for microswitches. These include 1) the compatibility of gallium; gallium can easily form alloys with most metals and cause embrittlement in certain metals; 2) the natural oxide skin on gallium surface increases the contact resistance; 3) the natural oxide skin in gallium exhibits high affinity to different substrates. Therefore, the microswitch systems need to be designed properly to avoid alloying of gallium, and more importantly, to prevent oxide adhesion in practical applications. Coating the gallium‐based liquid metal with Teflon helped to eliminate adhesion in microelectromechanical systems (MEMS) capacitive switch.^[^ ^174^ ^]^

In addition to Teflon, other systems such as glycerol solution, can also prevent the adhesion of liquid metal particles for sensors.^[^ ^175^ ^]^ Placing a liquid metal particle between two electrodes separated by a dielectric layer creates a capacitor. Depending on the location of the liquid metal particle, the contact area of the capacitor changes, and therefore the output capacitance alters accordingly.

The ability to bridge or break controllably two hemispheres of liquid metal offers a way to create a conductive switch similar to a transistor. Depending on the location with which potential was applied in a basic aqueous electrolytic solution, two liquid metal particles could separate or coalesce based on gradients in interfacial tension arising from the potential. Using this principle, a field‐control electrical switch (with transistor‐like properties) has been demonstrated for potential application as memory storage or logic and shape‐programmable circuitry.^[^ ^176^ ^]^

#### 4.3.7 Batteries

Lithium‐ion batteries are among the most promising candidates in the current rechargeable battery market. However, the volume expansion/contraction during the cycles of high‐capacity anodes, that is, silicon or tin, normally results in mechanical fracture and leads to inferior cycle performance. Therefore, a self‐healing property is highly desired for the system to further improve the cyclability. While gallium has a high theoretical capacity (769 mAh g^−1^), comparable to tin (990 mAh g^−1^), the benefit from its fluidic nature endows the anode composed of gallium alloy liquid metal nanoparticles with self‐healing properties to avoid the expansion/contraction‐induced cracking during the cycling process. A battery fabricated with gallium showed no obvious decay in capacity over 4000 cycles, which represents one of the best cyclable, all‐metal anodes.^[^ ^177^ ^]^ In addition, liquid metal can also combine with silicon materials to fabricate conductive‐additive‐free anode with excellent self‐healing properties.^[^ ^178^ ^]^

#### 4.3.8 Liquid Metal Marbles

Although the native oxide on liquid metals tends to adhere to many substrates, which causes issues in some applications, it also provides a facile way to coat/modify the liquid metal particles with a wide range of materials to engineer the surface properties. The concept of liquid metal marbles is evident when the liquid metal particles are coated with insulating particles (including Teflon or silica), semiconductor particles, that is, WO_3_, TiO_2_, MoO_3_, In_2_O_3_, or carbon nanotubes, by rolling the liquid metal particles over powders or submerging them into a colloidal suspension.^[^ ^77^ ^]^ The marble coating can endow liquid metal particles with mechanical stability and new functionality. For instance, liquid metal marbles could be used as active electronic junctions or heavy metal ion sensors.^[^ ^77^ ^]^ Specifically, liquid metals combined with WO_3_ coatings have the ability to induce bipolar electrochemically induced actuation.^[^ ^179^ ^]^ The WO_3_ coating can also behave as a photocatalyst that triggers a photochemical reaction in H_2_O_2_ the solution, generating oxygen bubbles to propel the marble.^[^ ^180^ ^]^ Moreover, with a coating of Teflon particles,^[^ ^181^ ^]^ liquid metal marbles possess high elasticity, mobility, and mechanical robustness, and when combined with a graphene coating, the resulted marbles are conductive.^[^ ^182^ ^]^

#### 4.3.9 Liquid Metal Motors/Robots

Liquid metal particles could also be employed as motors.^[^ ^183^ ^]^ When an aluminum flake is attached to a liquid metal particle and placed in an electrolyte (such as sodium hydroxide), hydrogen gas generated by the aluminum enables the movement of the particle; the direction and speed of such a self‐powered liquid metal motor can be controlled through electrical voltage.^[^ ^184^ ^]^ Interestingly, replacing aluminum with nickel induces dramatic hydrogen generation, leading to the intermittent jumping of liquid metal particles instead of swimming.^[^ ^185^ ^]^ In addition to liquid metal motors, coated with rigid shells, liquid metal particles could behave as vehicles for cargo delivery.^[^ ^185^ ^]^ Furthermore, it is also possible to actuate the liquid metal droplet with voltage as a wheeled robot. Displacement of the metal by potential‐induced surface tension gradients changes the center of gravity along a circular wheel, inducing torque that causes the wheel to rotate. Such a system operates outside a liquid environment, demonstrating the possibility to utilize liquid metal in more complex robotic systems.^[^ ^186^ ^]^

While the abovementioned liquid metal motors convert chemical energy to mechanical activity, there are also other strategies to induce the movement of liquid metal particles. Specifically: 1) In aqueous solution, liquid metal particles could “run” along metallic traces, that is, gold or silver, due to their tendency to wet and subsequently dissolve these metals. The speeds of such particles are remarkable ≈200 body lengths per second, while the running speed of Crangon shrimp, the fastest known aqueous creature, is ≈75 body lengths per second.^[^ ^187^ ^]^ 2). The ionic imbalance on liquid metal particles induces surface Marangoni flow to trigger self‐propulsion. Such an autonomous motion could be utilized for pumping or switching.^[^ ^188^ ^]^

#### 4.3.10 Optics

Liquid metal particles can be integrated into optical devices. For instance, liquid metal droplets can be dispensed on a substrate and then topped with a parylene/Teflon coated indium tin oxide glass slide as an area‐tunable micromirror.^[^ ^189^ ^]^ Based on electrowetting actuation, the radius of the micromirror and the normalized area can be tuned accordingly to change the relative reflected energy from 0% to 100% within 1 ms. Liquid metal particles can also be immobilized onto electrodes under an electric field to form 3D microstructures.^[^ ^190^ ^]^ The 3D electrodes can be fabricated with different aspect ratios and sizes, and they exhibit superior trapping efficiency of metal oxide nanoparticles compared to planar microelectrodes. Moreover, they can enhance the convective heat transfer within a microfluidic channel.^[^ ^190^ ^]^

Furthermore, gallium or gallium‐based alloys nanoparticles possess plasmonic resonance behavior while the nanoparticles could be located on substrates or in colloidal format.^[^ ^94^, ^191^, ^192^, ^193^, ^194^ ^]^ More interestingly, the plasmonic resonance of gallium nanoparticles is affected by the oxide skin on the surface of the nanoparticles, therefore, the plasmonic resonance is tunable through thermal oxidation to change the thickness of the gallium oxide without significant reduction of the plasmon resonance intensity. Plus, such particles could be utilized for surface‐enhanced Raman scattering substrate preparation.^[^ ^195^ ^]^

## 5 Challenges, Opportunities, and Open Questions

There are a number of remaining challenges and opportunities for liquid metal particles. Many were mentioned throughout this review, and some are mentioned below. The reader is also directed to a recent perspective article about opportunities of liquid metals in nanotechnology, which discusses reactivity and phase behavior (not discussed in this section).^[^ ^40^ ^]^

### 5.1 How to Improve the Conductivity of Liquid Metal Traces Formed from Particles?

While gallium has a very high electrical conductivity (3.4 × 10^6^ S m^−1^) compared to other room‐temperature liquid conductors, it is still one magnitude lower than copper. Liquid metal composites have an even lower conductivity (9.6 × 10^4^ S m^−1^).^[^ ^62^ ^]^ In these composites, liquid metal particles merge to form a percolated path through an elastomeric matrix. The lower conductivity may be attributed to the circuitous electrical path through the matrix as well as an increased amount of oxide due to the large surface areas of liquid metal droplets compared to bulk materials. Although higher conductivity is always desired in electronic devices to minimize losses, the conductivity of liquid metals is more than sufficient for devices such as speaker cables, interconnects, and other circuits. Yet, increasing conductivity can improve the efficiency of radio frequency related devices, especially at high frequency. Therefore, further improving the conductivity of liquid metal devices is of interest.

One promising approach is to combine liquid metal particles with other conductors, such as silver.^[^ ^196^ ^]^ In addition, recent work shows that platinum‐decorated carbon nanotubes can be mixed with liquid metal due to metal–metal wetting between the Pt and Ga.^[^ ^197^ ^]^

### 5.2 How do Two Liquid Metal Particles Merge?

When two liquid metal droplets contact each other under sufficient pressure, they merge. It is unclear by what mechanism this merging process takes place. More specifically, what happens to the oxide skin that was previously present at the contact between the two particles? Does the oxide migrate to the surface of the new particle? The fate of the oxide could impact the electrical and rheological properties of structures formed by mechanical sintering as well as 3D printing. Fundamentally understanding the merging process might help to design liquid metal devices with better electrical performance or engineered rheological properties.

### 5.3 How to Better Store Liquid Metal Particles?

It is possible to store colloidal liquid metal particles in solution after appropriately modifying their surfaces or when using surfactants, as detailed earlier. Yet, particle surface modification or the presence of surfactants might hinder the conductivity of the particles, and therefore, additional post‐processing steps that remove the surfactants/surface modification may be required. Moreover, gallium‐based liquid metal nanoparticles in aqueous solution tend to oxidize over time, forming gallium oxide hydroxide nanorods.^[^ ^87^ ^]^ Thus, it is critical to find a proper method to store liquid metal particles, especially in biological buffers for biomedical applications.^[^ ^118^ ^]^

### 5.4 How to Produce Nanoparticles with Narrow Distribution and High Concentration?

In terms of size distribution, studies show that the distribution of particles formed by sonication depends on the length of time of sonication,^[^ ^121^ ^]^ the power, and the chemical environment that surrounds the drops.^[^ ^85^ ^]^ Typical sizes range from 10 to 100 nm.^[^ ^62^, ^66^, ^70^, ^78^, ^121^ ^]^ Other methods, such as microfluidic flow focusing, produce almost perfectly monodisperse droplets but are limited to larger sizes (10–100 microns),^[^ ^103^, ^104^, ^108^, ^109^, ^110^, ^111^, ^112^ ^]^ although the size can be controlled by applying a potential to the metal. Shear driven formation of particles can produce a wide range of sizes, from 10 nm to 10s of microns.^[43]^

In certain applications, such as drug delivery, additional post‐processing, including centrifuging or filtering, needs to be conducted to narrow the size distribution. As a result, the concentration of the nanoparticles of an appropriate size may be relatively low. The highest concentration of liquid metal particles after 72 h is reported to be only ≈0.2 mm.^[^ ^116^ ^]^ Narrowing the distribution is important for applications in which particle size affects performance, such as composites. Particles with controlled diameter distribution at high concentrations will provide better control over the properties of composites that use such particles.

### 5.5 Best Practices for Liquid Metal Contacts?

Gallium forms conductive composites that are soft and stretchable. These conductors often require external electrical contacts, yet gallium is known to embrittle many metals, which creates a challenge. For example, Ga rapidly attacks aluminum and causes it to embrittle. In our lab, we utilize copper wires to contact liquid metal without issue, but this has not been studied to prove long term durability. Recent work has shown it possible to use barriers between the liquid metal and contacts, such as thin layers of graphene that are both physical barriers, yet electrical conductors. Alternatively, other conductive composites, such as aligned Ni particles, can help create conductive contacts.^[^ ^198^ ^]^ Finding ways to directly contact liquid metals may enable them to be used as solders, wires, and interconnects in stretchable devices.

## 6 Conclusions

Gallium‐based liquid metals possess a unique combination of fluidic and metallic properties. Unlike mercury, gallium‐based liquid metals exhibit low toxicity. The ability to deform the conductive liquid metal combined with the reactivity of gallium opens up many new opportunities. Gallium‐based liquid metal particles feature some unique characteristics: including size‐tunability, shape‐reconfigurability, a core–shell structure, and the ability to mechanically sinter and self‐heal. A variety of methods, including 3D printing, molding, microfluidic production, shearing, or sonication, can fabricate particles over a range of sizes. The method of choice depends typically on the size of the particle one wants to produce. Liquid metal particles can be utilized for a variety of applications, including (but not limited to) soft electronics, drug delivery, microfluidics, energy‐harvesting, batteries, motors/robots, or composites. The unique attributes, combined with the diverse application space, makes the future of liquid metal particles bright.

## Conflict of Interest

The authors declare no conflict of interest.
